# MiR-125b-5p and miR-100-5p as Biomarkers and therapeutic targets for the prevention of particulate matter-induced non-smoker lung cancer

**DOI:** 10.1371/journal.pone.0337805

**Published:** 2025-12-02

**Authors:** Moe Thi Thi Han, Tay Zar Myo Oo, Busayamas Chewaskulyong, Sakorn Pornprasert, Kanyamas Choocheep, Khanittha Punturee, Warunee Kumsaiyai, Yupanun Wuttiin, Sawitree Chiampanichayakul, Ratchada Cressey

**Affiliations:** 1 Department of Medical Technology, Faculty of Associated Medical Sciences, Chiang Mai University, Chiang Mai, Thailand; 2 Department of Internal Medicine, Faculty of Medicine, Chiang Mai University, Chiang Mai, Thailand; 3 Cancer Research Unit, Department of Medical Technology, Faculty of Associated Medical Sciences, Chiang Mai University, Chiang Mai, Thailand; University of Catania, ITALY

## Abstract

Non-smoking-related lung cancer is increasingly associated with environmental factors such as particulate matter (PM) exposure. Using deep small RNA sequencing, we identified distinct miRNA expression patterns in lung cancer patients compared to non-cancer controls, stratified by smoking status. Notably, hsa-miR-125b-5p and hsa-miR-100-5p were significantly downregulated in non-smoking lung cancer patients. Pathway enrichment analysis revealed smoking amplifies pathways related to glycan biosynthesis, signal transduction, and transcriptional regulation, while non-smoking lung cancer is characterized by immune dysfunction and metabolic alterations, including oxidative phosphorylation and natural killer cell cytotoxicity. Validation in a larger cohort using quantitative RT-PCR confirmed the suppression of miR-125b-5p and miR-100-5p in non-smoking lung cancer patients. Additionally, miR-203a and miR-199a-3p were identified as potential biomarkers for lung cancer, independent of smoking status. Chronic PM exposure in primary bronchial/tracheal epithelial cells initially elevated miR-125b-5p and miR-100-5p expression, but prolonged exposure suppressed these miRNAs while increasing their target genes, *TXNRD1* and *HOXA1*, suggesting stress-induced dysregulation. Functional studies using miRNA mimics demonstrated that miR-125b-5p and miR-100-5p suppress PM-induced cancer cell mobility and colony formation, with miR-125b-5p exhibiting broader effects. These findings underscore the critical roles of miR-125b-5p and miR-100-5p in PM-associated lung cancer progression and their potential as biomarkers and therapeutic targets. This study highlights distinct mechanisms of lung carcinogenesis in smokers and non-smokers, providing a foundation for targeted interventions in PM-associated lung cancer.

## 1. Introduction

The increasing global prevalence of lung cancer in non-smokers highlights the impact of environmental factors such as radon and air pollution. Across regions, evidence reveals varying rates of lung cancer cases among non-smokers, particularly with a significant rise in mortality linked to smoking in low- and middle-income nations [1]. Air pollution in Northern Thailand poses a notable public health concern [2]. Research has identified the link between air pollution, specifically fine particulate matter (PM2.5), and the onset of lung cancer [3–5], as PM2.5 capable of penetrating deep into the lungs thus may induce inflammation and contribute to the initiation and advancement of cancerous cells. It has been demonstrated that the risk of lung cancer in non-smokers increases in a dose-dependent manner with the concentration of ambient air pollutants [6].

MicroRNAs (miRNAs) have shown great potential as biomarkers for understanding the health effects of air pollution. These small RNA molecules are essential for cellular function as they regulate gene expression. They fine-tune protein levels, control cell differentiation and development, influence the cell cycle, apoptosis, and immune response [7]. Dysregulation of miRNAs is associated with diseases [8–10], making them important in understanding cellular functions and disease mechanisms. miRNAs can reflect cellular responses to environmental stressors, and changes in their expression patterns have been observed in individuals exposed to air pollution [11–14], suggesting a potential link between air quality and miRNA alterations. Investigation of the miRNA expression profile in serum, particularly its distinctions between individuals with lung cancer and a control group, holds considerable significance for lung cancer screening.

In this study, we analyzed serum miRNA expression profiles in lung cancer patients, stratified by smoking history, and compared them to non-cancer controls. Our findings identified distinct miRNA expression patterns, particularly in non-small cell lung cancer (NSCLC) patients without a history of smoking. Further in vitro experiments demonstrated a correlation between particulate matter exposure and these miRNA alterations. These results emphasize the role of miRNAs as biomarkers and potential therapeutic targets for PM-induced lung cancer. They also highlight the urgency of addressing air pollution in Northern Thailand to mitigate its impact on public health.

## 2. Materials and methods

### 2.1. Study population and sample procession

Participants were prospectively recruited for this study, with blood samples collected from patients newly diagnosed with non-small cell lung cancer (NSCLC) at Maharaj Nakorn Chiang Mai Hospital and from healthy individuals undergoing routine health check-ups at the Clinical Service Center, Faculty of Associated Medical Sciences, Chiang Mai University, between 25/05/2021–24/05/2022 and 23/05/2023–18/05/2024. Inclusion criteria for the NSCLC group were: (1) newly diagnosed and histologically confirmed non-small cell lung cancer, (2) no prior cancer treatment (chemotherapy, radiotherapy, or immunotherapy), and (3) age ≥ 18 years. Exclusion criteria included a history of other malignancies, chronic inflammatory, autoimmune, or metabolic diseases (e.g., rheumatoid arthritis, diabetes mellitus), current infection or fever, pregnancy or breastfeeding, and recent major surgery or trauma (within three months).

For the non-cancer control group, inclusion criteria were: (1) age- and sex-matched individuals attending routine health check-ups, and (2) no clinical history or symptoms suggestive of lung disease or malignancy. Exclusion criteria mirrored those of the NSCLC group.

Written informed consent was obtained from all participants prior to their enrollment in the study. The consent process was conducted following ethical guidelines, with written forms signed in the presence of a witness. Only adults were included in the study, and proxy consent was not applicable. Ethical approval (Approval Number: NONE-2564–08207) was granted by the Human Ethics Committee, Faculty of Medicine, Chiang Mai University, Thailand. The study adhered to the principles outlined in the Declaration of Helsinki.

Blood samples (5–10 mL) were collected from NSCLC patients before the initiation of any treatment and from non-cancer controls during routine check-ups. Serum was separated from the collected blood samples and stored at −80°C until analysis. Total miRNA was extracted from the serum using the NucleoSpin serum miRNA extraction kit (Macherey-Nagel, Germany) following the manufacturer’s instructions. As the serum samples were processed without prior removal of exosomes, the extracted miRNA comprises both the soluble fraction and exosome-associated miRNA. Extracted miRNA samples were aliquoted and stored at −20°C for subsequent analyses.

### 2.2. Small RNA sequencing

miRNA was extracted from the serum of nine subjects, including three individuals with non-small cell lung cancer (NSCLC) with a smoking history, three NSCLC patients without a smoking history, and three non-cancer controls (two non-smokers and one smoker). The extracted miRNA underwent small RNA deep sequencing and data analysis at Vishuo Biomedical Pte. Ltd., as previously described [15]. In brief, approximately 10 ng of small RNA was used for library preparation, involving adaptor ligation, cDNA synthesis, and PCR amplification. The libraries were then multiplexed and sequenced on the Illumina system.

Raw sequencing reads underwent quality control using Trimmomatic (v0.30) to remove adapter sequences, trim low-quality bases (quality score <20), and discard reads shorter than 18 bp [16]. miRNA identification was performed using miRDeep2 [17], a computational tool for discovering known and novel miRNAs from deep sequencing data. This allowed for the retrieval of all identified miRNAs along with their expression profiles. Differential expression analysis of miRNAs was conducted using the DESeq/DESeq2 Bioconductor package [18], which employs a model based on the negative binomial distribution. Adjustments for multiple testing were applied using the Benjamini and Hochberg method to control the false discovery rate. miRNAs with p < 0.05 and fold change ≥ 2 were considered significantly differentially expressed. For RT-qPCR validation, the subset of miRNAs with p < 0.05 and fold changes greater than 6.0 were selected.

Functional enrichment analysis was conducted to explore the biological significance of DEGs and miRNA target genes. KEGG (Kyoto Encyclopedia of Genes and Genomes) pathway enrichment analysis was performed using in-house scripts, classifying DEGs into major biological categories, including organismal systems, metabolism, human diseases, environmental information processing, and cellular processes. Enrichment results were visualized using bar and dot plots, where dot size represented the number of genes, and color indicated statistical significance (Q-value). miRNA-mRNA interactions were predicted using miRanda [19] for animal models. The predicted target genes were further analysed for pathway involvement and regulatory networks. Comparisons were conducted for non-cancer control vs NSCLC patients with smoking history, non-cancer control vs NSCLC patients without smoking history, and NSCLC patients with smoking history vs. NSCLC patients without smoking history to investigate cancer-related pathways.

### 2.3. Quantitative RT-PCR (RT-qPCR)

For quantification of miRNA expression level, cDNA synthesis was carried out using a polyadenylation-based method [20], followed by qPCR using iTaq™ Universal SYBR® Green supermix on a CFX96 Touch™ Real-Time PCR Detection System (Bio-Rad). miR-484 was used as the endogenous control [21]. The relative expression of miRNAs was determined using the equation 2-ΔCT, where ΔCT represents the difference between the average cycle threshold (CT) of the specific miRNA and the average CT of the endogenous reference miRNA in cancer patients or non-cancer controls.

Additionally, the expression of *HOXA1* and *TXNRD1*, known target genes of miR-100-5p and miR-125b-5p, respectively, was quantified to assess the downstream effects of PM exposure. Following RNA extraction and cDNA synthesis, qPCR was performed using SYBR Green-based detection, with 18S rRNA as the endogenous reference gene.The primers used in this study were synthesized by Macrogen (Seoul, South Korea), and their sequences are as follows:

**Table:** 

Name of Primers/ Probes	Sequence (5’ to 3’)
miR-125b-5p:	5’-AACCACTTCCCTGAGACCCTAAC-3’
miR-100-5p:	5’-AACAAGAACCCGTAGATCCGAAC-3’
miR-203a-3p:	5’-AACCGGGTGAAATGTTTAGGACC-3’
miR-107:	5’-AACAGAAGCAGCATTGTACAGGG-3’
miR-9-5p:	5’-ACGCCGTCTTTGGTTATCTAGCT-3’
miR-199a-3p:	5’-AACACGCACAGTAGTCTGCAC-3’
Universal reverse primer:	5’-AACACGTGTGAGGTAGTAGGTTGTA-3’
*HOXA1* Forward:	5’-CGGAACTGGAGAAGGAGTTC-3’
*HOXA1* Reverse:	5’-TTCACTTGGGTCTCGTTGAG-3’
*TXNRD1* Forward:	5’-CCACTGGTGAAAGACCACGTT-3’
*TXNRD1* Reverse:	5’-AGGAGAAAAGATCATCACTGCTGAT-3’
18s rRNA Forward:	5′-AGGAATTGACGGAAGGGCAC-3′
18S rRNA Reverse:	5′-GTGCAGCCCCGGACATCTAAG-3′

### 2.4 *In vitro* investigation of urban particulate matter exposure on miRNA expression

The impact of PM on the biological function of human primary bronchial/tracheal epithelial cells (PCS-300–010, ATCC) was explored through *in vitro* investigations. Cells were cultured in airway epithelial cell basal medium (ATCC PCS-300–030) supplemented with a bronchial/tracheal epithelial cell growth kit (ATCC PCS-300–040). The kit contains the following supplements at the specified final concentrations: HSA (500 mg/mL), linoleic acid (0.6 mM), lecithin (0.6 mg/mL), L-glutamine (6 mM), extract P (0.4%), epinephrine (1.0 mM), transferrin (5 mg/mL), T3 (10 nM), hydrocortisone (5 mg/mL), rh EGF (5 ng/mL), and rh insulin (5 mg/mL).

The urban particulate matter (UPM; SRM 1648a) were obtained from the National Institute of Standards and Technology in the United States. These particles, with an average diameter of 5.85 μm, contained 21 metal elements, 4 non-metallic metals, 21 polycyclic aromatic hydrocarbons (PAHs), and 7 polychlorinated biphenyl congeners. Human primary bronchial/tracheal epithelial cells were seeded at a density of 5 × 10^4 cells per well in a 6-well plate and exposed to PM concentrations of 50 µg/mL and 100 µg/mL for a duration of 1–7 days. The control group was cultured without UPM treatment. Subsequently, RNA (miRNA and mRNA) was extracted from both the control group and the PM-treated groups, and the samples were stored at −20 °C until subjected to further experiments.

### 2.5 Extraction of mRNA and miRNA from cultured cells

miRNA and mRNA extraction were conducted using the Nucleospin miRNA kit (Cat no. 740971, Macherey-Nagel) following the manufacturer’s instructions. 300 µL of Buffer ML was added to the cells and vortexed for 5 minutes at room temperature. The lysate was then loaded into a NucleoSpin® Filter column and centrifuged for 1 minute at 11,000 x g to remove cell debris. Subsequently, 150 µL of 96–100% ethanol was added to the flowthrough to optimize binding conditions. This mixture was left to stand for 5 min at room temperature. The resulting solution was loaded into a NucleoSpin® RNA column and centrifuged for 1 min at 11,000 x g. After collecting the flowthrough containing small RNA, the NucleoSpin® RNA column was placed in a new collection tube (2mL) and filled with 350 µL of Buffer MDB to extract large RNA. To eliminate any contaminating DNA, 100 µL of rDNase was added to the column and incubated for 15 min at room temperature.

The flowthrough containing small RNA was mixed with 300 µL Buffer MP and centrifuged for 3 min at 11,000 x g to remove precipitated proteins. The remaining protein was further eliminated from the supernatant by filtration through a NucleoSpin® Protein Removal Column. The resulting flowthrough was then mixed with 800 µL Buffer MX, and the mixture containing small RNA was loaded into a second NucleoSpin® RNA Column. Ultimately, both columns, each containing small (miRNA) and large (mRNA) RNA, underwent washing with Wash Buffers MW1 and MW2 before being eluted with 30 µL RNase-free H2O. The extracted RNAs were stored at −20°C until further analysis. RNA purity and integrity were assessed using NanoDrop spectrophotometry, and all samples met the standard quality criteria (A260/A280 ratio between 1.8 and 2.1). To minimize potential degradation, reverse transcription and qPCR reactions were performed within 24 hours of extraction. Gene expression levels were normalized using 18S rRNA, a widely accepted and stable internal control.

### 2.6 Transfection and cell viability assay

To examine the effect of UPM and miRNA mimics on cell viability of lung cancer cells, human lung adenocarcinoma A549 cells (obtained from the American Type Culture Collection, ATCC) were transfected with miR-100-5p mimics, miR-125b-5p mimics (mirVana™ miRNA Mimic, Thermo Fisher), or a negative control mimic (mirVana™ Negative Control, Thermo Fisher) at final concentrations of 10 nM and 50 nM. Transfection complexes were prepared using RNAiMAX transfection reagent (Invitrogen, Thermo Fisher Scientific) following the manufacturer’s protocol. Mimics and RNAiMAX reagent were separately diluted in incomplete Dulbecco’s Modified Eagle Medium (DMEM) and incubated at room temperature for 20 minutes for complex formation. The transfection mixture (20 µL) was then added to wells containing 100 µL of A549 cells, with or without PM pre-treatment, maintaining a 1:5 transfection mix-to-cell culture media ratio.

For transfection in a 96-well plate, miR-100-5p mimics, miR-125b-5p mimics (mirVana™ miRNA Mimic, Thermo Fisher), and negative control (mirVana™ Negative Control, Thermo Fisher) were prepared to achieve final concentrations of 10 nM and 50 nM. Transfection complexes were assembled using RNAiMAX transfection reagent (Invitrogen, Thermo Fisher Scientific) following the manufacturer’s protocol. Mimics and RNAiMAX reagent were each diluted in incomplete Dulbecco’s Modified Eagle Medium (DMEM) and incubated at room temperature for 20 minutes for complex formation. The transfection mixture (20 µL) was then added to wells containing 100 µL of cells with and without the pre-treatment of PM, maintaining a 1:5 transfection mix-to-cell culture media ratio.

Cell viability following transfection was assessed using the MTT (3-(4,5-Dimethylthiazol-2-yl)-2,5-diphenyltetrazolium bromide) assay. After the desired incubation period post-transfection, 20 µL of MTT solution was added to each well and incubated at 37°C for 4 hours to allow formazan crystal formation. To dissolve the crystals, 100 µL of dimethyl sulfoxide (DMSO) (Sigma Aldrich, cat.no., D8418), was added to each well, followed by incubation on a shaker for 30 minutes in the dark. Absorbance was measured at 570 nm using a microplate reader.

### 2.7 Anchorage independent growth assay

The impact of miR-100-5p and miR-125b-5p mimics on PM-induced anchorage-independent growth was assessed using a soft agar colony formation assay. A549 and H1299 lung cancer cells were transfected with miR-100-5p, miR-125b-5p, or non-target control mimics (10 nM or 50 nM) using Lipofectamine™ RNAiMAX, following the manufacturer’s protocol. For the base layer, 25 µL of pre-warmed (37°C) 2 × DMEM supplemented with 20% FBS was combined with an equal volume of 0.8% agarose (pre-warmed to 56°C) and plated in a 96-well microplate. After solidification, a cell suspension containing 1.5 × 10³ A549 cells, pre-incubated with the transfection complex and exposed to UPM (final concentration: 1.0 µL/mL), was mixed with 30 µL of 2 × DMEM supplemented with 20% FBS and an equal volume of 0.6% agarose before being layered onto the base. To prevent desiccation, a feeder layer composed of 25 µL of 2 × DMEM with 20% FBS and 25 µL of 0.8% agarose was added.

Plates were incubated at 37°C in a 5% CO₂ atmosphere for two weeks. Each condition was performed in triplicate. Colony formation was quantified using the alamarBlue assay, and fluorescence was measured at 570 nm using a Synergy™ 4 Multi-Detection Microplate Reader (BioTek, MA, USA).

### 2.8 Cell migration assay

The migration ability of A549 and H1299 cells following transfection was assessed using a Transwell® system in a 6-well Boyden Chamber (8.0 µm pore polycarbonate membrane insert; Corning, cat. no. 3428). For transfection, cells were first seeded at a density of 1.0 × 10⁶ per well in serum-free DMEM into the upper chamber of each insert. The transfection complex containing miR-100-5p mimics, miR-125b-5p mimics (mirVana™ miRNA Mimic, Thermo Fisher), or negative control mimics was then added at final concentrations of 10 nM or 50 nM. Immediately after transfection, PM (1.0 µg/mL) was added to the upper chamber to ensure continuous exposure during the assay.

Following transfection, 20% FBS-containing medium was added to the lower chamber as a chemoattractant. Cells were incubated at 37°C in a CO₂ incubator for 20 hours to allow migration. After incubation, non-migrated cells were removed from the upper chamber, and migrated cells were fixed with methanol, stained with 0.2% crystal violet, and visualized under a light microscope. For quantification, stained cells were extracted in 200 µL of DMSO, and optical density was measured at 595 nm using an Emax Plus microplate reader (Molecular Devices, California, USA).

### 2.9 Statistical analysis

All statistical analyses were performed using SPSS (version 17, IBM Corp., Armonk, NY). The distribution of relative miRNA expression values (normalized to miR-484) was first examined using the Shapiro–Wilk test and visual inspection of histograms. For group comparisons, non-parametric testing was applied. Specifically, the Mann–Whitney U test was used to compare miRNA expression levels between lung cancer patients and non-cancer controls, and subgroup comparisons were performed according to smoking history. In addition, binary logistic regression analyses were conducted using log-transformed relative expression values to assess the independent association between miRNA expression and cancer status (case/control) while adjusting for potential confounders including age, sex, and smoking status. A two-tailed p-value < 0.05 was considered statistically significant.

## 3 Results

### 3.1 Identification of differentially expressed circulating miRNAs through small RNA deep sequencing

To account for potential smoking-related effects, the non-cancer control group included both smokers and non-smokers as a combined reference. This approach allowed us to focus on lung cancer-specific miRNA alterations, particularly those associated with PM exposure in non-smokers, while recognizing that smokers may experience a combined impact of cigarette smoke and PM exposure.

**Fig 1:**
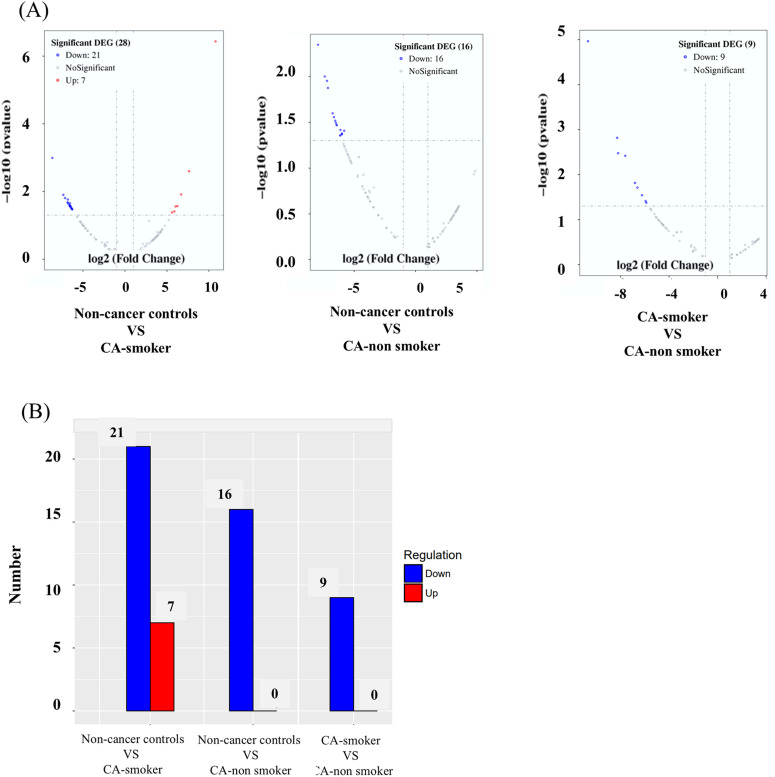
Differentially expressed genes (DEGs) in different comparisons of NSCLC patients and non-cancer controls. (CA-smoker, NSCLC patients with smoking history; CA-non-smoker, NSCLC patients without smoking history). **a)** Volcano plots showing the distribution of DEGs for three comparisons: CA-smoker vs. CA-non-smoker, non-cancer control vs. CA-non-smoker, and non-cancer control vs. CA-smoker (CA-smoker, NSCLC patients with smoking history; CA-non-smoker, NSCLC patients without smoking history). The x-axis represents the log2 fold change (FC) in gene expression, while the y-axis represents the –log10(p-value). Genes with significant downregulation are shown in blue, upregulated genes in red, and non-significant genes in gray. The number of significant DEGs in each comparison is indicated in the legend. (b) Bar graph summarizing the number of significantly up-regulated (red) and down-regulated (blue) DEGs across the three comparisons. The numbers on top of each bar correspond to the total count of DEGs in each group. The CA-smoker vs. CA-non-smoker comparison identified 9 down-regulated DEGs, while non-cancer controls vs. CA-non-smoker comparison revealed 16 down-regulated DEGs. The non-cancer controls vs. CA-smoker comparison had the highest number of DEGs, with 21 down-regulated and 7 up-regulated genes.

The analysis of deep sequencing data revealed a distinct set of miRNAs significantly altered based on a fold change greater than 2 and a p-value less than 0.05 (Fig 1a). When comparing non-cancer controls to NSCLC patients with smoking history (CA-smoker), 21 miRNAs were significantly downregulated, and 7 miRNAs were upregulated. In contrast, 16 miRNAs were significantly downregulated in NSCLC patients without smoking history (CA-non-smoker). Within lung cancer patients, 9 miRNAs were significantly downregulated in NSCLC patients without smoking history compared to NSCLC patients with smoking history (Fig 1a). Table 1 present the differentially expressed novel and known circulating miRNAs in cancer patients compared to non-cancer controls. Furthermore, Fig 1b delineates the unique sets of miRNAs that exhibit differential expression in NSCLC patients with smoking history, with no smoking history, and those shared between both groups of NSCLCs.

**Table 1:** List of miRNAs identified by small RNA deep sequencing to be differentially expressed in lung cancer patients with and without smoking history compared to non-cancer controls, as well as comparisons between lung cancer patients with different smoking histories.

I. Non-cancer controls vs NSCLS patients with smoking history							
Increased miRNAs	Fold-change	P value	FDR-BH	Decreased miRNAs	Fold-change	P value	FDR-BH
1. NovelmiRNA-285	10.7	3.6E-07	1.0E-05	1. hsa-let-7f-5p	−8.6	0.001	0.014
2. NovelmiRNA-194	7.6	0.002	0.023	2. hsa-miR-203a-3p	−7.3	0.012	0.036
3. hsa-miR-9-5p	6.6	0.012	0.036	3. NovelmiRNA-35	−7.1	0.015	0.036
4. NovelmiRNA-297	6.2	0.026	0.036	4. hsa-miR-199b-3p	−6.8	0.021	0.036
5. NovelmiRNA-124	6.0	0.027	0.036	5. hsa-miR-199a-3p	−6.8	0.021	0.036
6. NovelmiRNA-74	5.8	0.038	0.040	6. hsa-miR-107	−6.7	0.017	0.036
7. hsa-miR-5004-3p	5.6	0.041	0.041	7. hsa-miR-103a-3p	−6.7	0.022	0.036
				8. hsa-miR-30a-5p	−6.6	0.026	0.036
				9. hsa-miR-374b-5p	−6.5	0.025	0.036
				10. hsa-miR-200a-3p	−6.5	0.027	0.036
				11. hsa-miR-128-1-3p	−6.5	0.022	0.036
				12. hsa-miR-22-3p	−6.5	0.026	0.036
				13. NovelmiRNA-273	−6.4	0.027	0.036
				14. hsa-let-7d-5p	−6.4	0.029	0.036
				15. hsa-miR-409-3p	−6.3	0.030	0.036
				16. NovelmiRNA-31	−6.3	0.032	0.036
				17. NovelmiRNA-55	−6.3	0.032	0.036
				18. NovelmiRNA-62	−6.3	0.032	0.036
				19. NovelmiRNA-145	−6.3	0.032	0.036
				20. NovelmiRNA-277	−6.3	0.032	0.036
				21.hsa-miR-27a-3p	−6.2	0.033	0.036
**II. Non-cancer controls vs NSCLS patients without smoking history**							
Increased miRNAs	Fold-change	P value	FDR-BH	Decreased miRNAs	Fold-change	P value	FDR-BH
–	–	–	–	1. hsa-miR-100-5p	−7.9	0.004	0.043
				2. hsa-miR-107	−7.4	0.009	0.043
				3. hsa-miR-125b-5p	−7.2	0.011	0.043
				4. hsa-miR-128-1-3p	−7.1	0.013	0.043
				5. hsa-miR-203a-3p	−6.7	0.025	0.043
				6. NovelmiRNA-273	−6.6	0.027	0.043
				7. hsa-miR-200b-3p	−6.5	0.030	0.043
				8. NovelmiRNA-35	−6.4	0.032	0.043
				9. hsa-miR-101-3p	−6.4	0.033	0.043
				10. hsa-miR-101–2-3p	−6.4	0.033	0.043
				11. hsa-miR-128-2-3p	−6.1	0.038	0.043
				12. hsa-miR-143-3p	−5.8	0.039	0.043
				13. hsa-miR-423-3p	−6.0	0.041	0.043
				14. hsa-miR-186-5p	−5.9	0.042	0.043
				15. hsa-miR-199b-3p	−6.1	0.043	0.043
				16. hsa-miR-199a-3p	−6.1	0.043	0.043
**III. NSCLS patients with no smoking history vs NSCLS patients with smoking history**							
Increased miRNAs	Fold-change	P value	FDR-BH	Decreased miRNAs	Fold-change	P value	FDR-BH
**–**	–	–	–	1. NovelmiRNA-285	−10.6	1.0E-05	9.7E-05
				2. NovelmiRNA-194	−8.2	0.001	0.006
				3. NovelmiRNA-297	−8.2	0.003	0.008
				4. hsa-miR-100-5p	−7.6	0.003	0.008
				5. hsa-miR-25-3p	−6.8	0.015	0.027
				6. hsa-miR-125b-5p	−6.6	0.019	0.029
				7. hsa-miR-30b-5p	−6.2	0.028	0.036
				8. hsa-miR-205-5p	−5.9	0.038	0.041
				9. hsa-miR-629-5p	−5.8	0.0419	0.041

FDR-BH = FDR-corrected p-values (Benjamini-Hochberg method)

**Fig 2:**
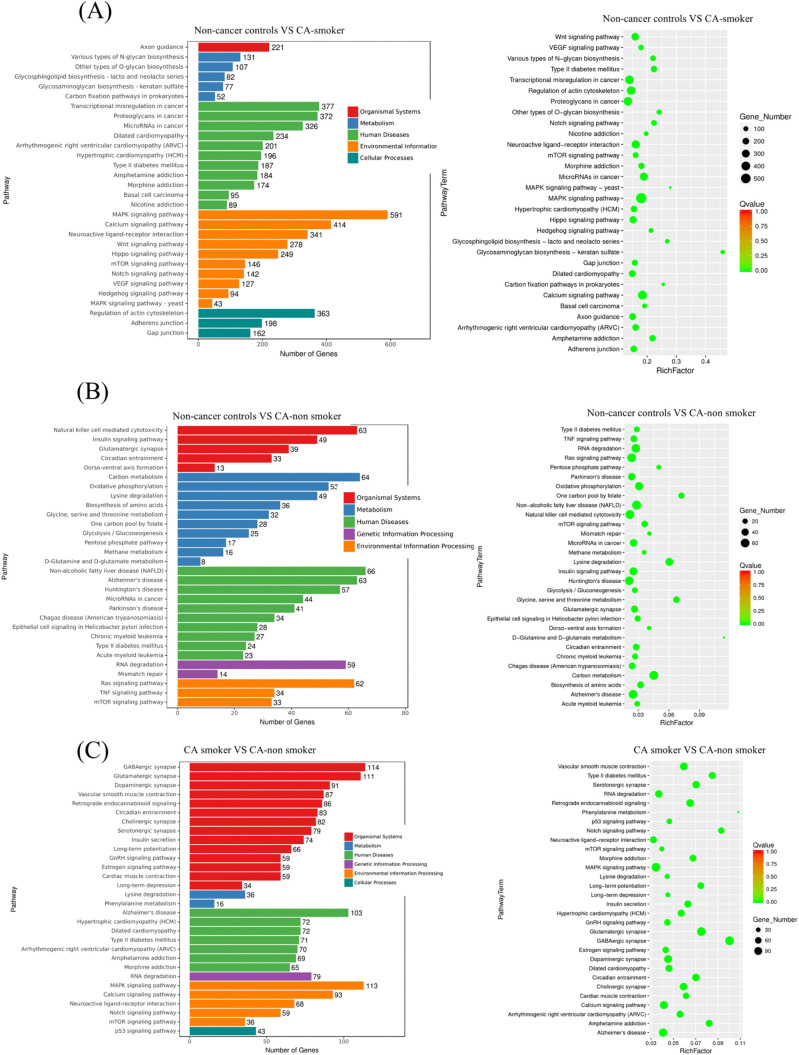
Illustrates KEGG pathway annotations of differentially expressed miRNA target genes. (a) depicts the KEGG enrichment histogram comparing NSCLC patients with smoking history to non-cancer controls, (b) shows the same comparison for NSCLC patients without smoking history versus non-cancer controls. (c) presents the comparison between NSCLC patients with smoking history to NSCLC patients without smoking history. In the histogram, the x-axis represents the number of differentially expressed genes, and the y-axis displays the names of the KEGG pathways. In the scatter plot, the x-axis indicates the rich factor, and the y-axis specifies the KEGG pathways. Dot size correlates with the number of differential miRNA target genes, while color indicates different Q value ranges. Higher rich factors denote greater enrichment, while smaller Q values signify more significant enrichment.

Subsequently, target genes of differentially expressed miRNAs were annotated with the Kyoto Encyclopedia of Genes and Genomes (KEGG pathway). Rich factors measured the degree of pathway enrichment, with bubble sizes representing the number of differentially expressed genes (DEG). Fig 2a-c displays the top 30 significantly enriched pathways. Comparison between non-cancer controls and NSCLC patients with smoking history, pathways enriched include metabolic pathways (e.g., glycosaminoglycan and N-glycan biosynthesis), transcriptional mis-regulation in cancer, and MAPK and Wnt signaling pathways, suggesting critical alterations in glycan metabolism, signal transduction, and transcriptional regulation. These changes highlight smoking-associated pathways in cancer development, with significant genes involved in human disease pathways, environmental processing, and cellular processes.

Conversely, comparison between non-cancer controls and NSCLC patients without smoking history, pathways exhibited enrichment include natural killer cell-media ted cytotoxicity, insulin signaling, and oxidative phosphorylation, indicating immune dysregulation, altered metabolic processes, and mitochondrial stress. Additionally, pathways like circadian entrainment and carbon metabolism were enriched, suggesting systemic impacts beyond cancer-specific pathways.

Finally, comparison between NSCLC patients with smoking history and patients with no smoking history, enriched pathways in smokers are synaptic signaling (e.g., glutamatergic and dopaminergic synapse pathways), metabolic dysregulation (e.g., lysine degradation, phenylalanine metabolism), and mitochondrial pathways (oxidative phosphorylation). NSCLC patients without smoking history showed relatively fewer enriched cancer-specific pathways, indicating distinct biological impacts of smoking on cancer progression.

Overall, Fig 2 demonstrates significant pathway differences between non-cancer controls and NSCLC patients, stratified by smoking status. Smoking amplifies pathways involved in glycan biosynthesis, signal transduction, and transcriptional regulation, while NSCLC patients without smoking history exhibit immune and metabolic dysregulation. These insights emphasize the role of smoking as a modifier of cancer-associated molecular pathways and the distinct mechanisms driving cancer in non-smokers.

### 3.2. Determination of serum miRNA expression level in a larger cohort of clinical samples using quantitative RT-PCR

**Fig 3:**
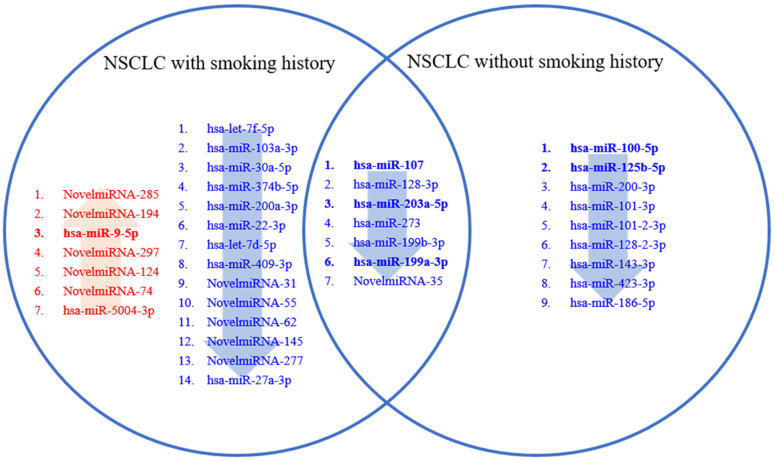
Venn diagram illustrates the list of miRNAs identified by small RNA deep sequencing as differentially expressed in NSCLC patients with smoking history, NSCLC patients without smoking history, and those commonly altered in both groups compared to non-cancer controls.

Based on significant changes observed in miRNAs from deep sequencing results (Table 1), we conducted validation using quantitative RT-PCR in an expanded sample set of 78 lung cancer patients and 50 non-cancer controls (Table 2). Fig 3 illustrates the lists of miRNAs differentially expressed in NSCLC patients with and without smoking history compared to non-cancer controls, as well as those commonly altered in both groups. These findings suggest the potential utility of these miRNAs as biomarkers and therapeutic targets tailored to patient subgroups based on smoking history. Our focus was on miR100-5p and miR125b-5p, comparing non-cancer controls to NSCLC patients without a smoking history (CA non-smoker). These two miRNAs were among the most significantly reduced in CA non-smokers, suggesting a potential role in lung cancer development independent of tobacco exposure. Furthermore, previous studies have reported their critical roles in lung cancer [22,23]. Additionally, we assessed miR9-5p, which showed significant differences between non-cancer controls and NSCLC patients with smoking history (CA smokers). We also examined miR-203-3p, miR-107, and miR-199a-3p, as they were significantly altered across NSCLC patients both with and without smoking (Fig 3). Furthermore, mTOR signaling was identified as a key pathway significantly affected in both smoker and non-smoker NSCLC patients. All three selected miRNAs (miR-203-3p, miR-107, and miR-199a-3p) have been reported to be directly or indirectly linked to mTOR regulation, further supporting their selection [24–26].

**Table 2:** Characteristic of NSCLC patients and non-cancer controls.

RNA sequencing set	Lung cancer (n = 6)		Control	
	**Non-smoker (n = 3)**	**Smoker** **(n = 3)**	**Non-smoker (n = 2)**	**Smoker (n = 1)**
Gender				
Male (n (%))	1 (33.33%)	2 (66.67%)	0 (0.0%)	1 (33.33%)
Female (n (%))	2 (66.67%)	1 (33.33%)	2 (66.67%)	0 (0.0%)
Age				
Mean ± SD	56.67 ± 8.21	64.74 ± 10.26	57 ± 1.00	59
**RT-PCR set**	**Lung cancer (n = 78)**		**Controls (n = 50)**	
	**Non-smoker (n = 35)**	**Smoker (n = 43)**	**Non-smoker (n = 42)**	**Smoker (n = 8)**
Gender				
Male (n (%))	6 (14.3%)	33 (76.7%)	15 (35.7%)	6 (75.0%)
Female (n (%))	29 (85.7%)	10 (23.3%)	27 (64.3%)	2 (25.0%)
Age				
Mean ± SD	63.63 ± 11.09	64.74 ± 10.26	62.32 ± 7.73	60.66 ± 9.62

**Fig 4:**
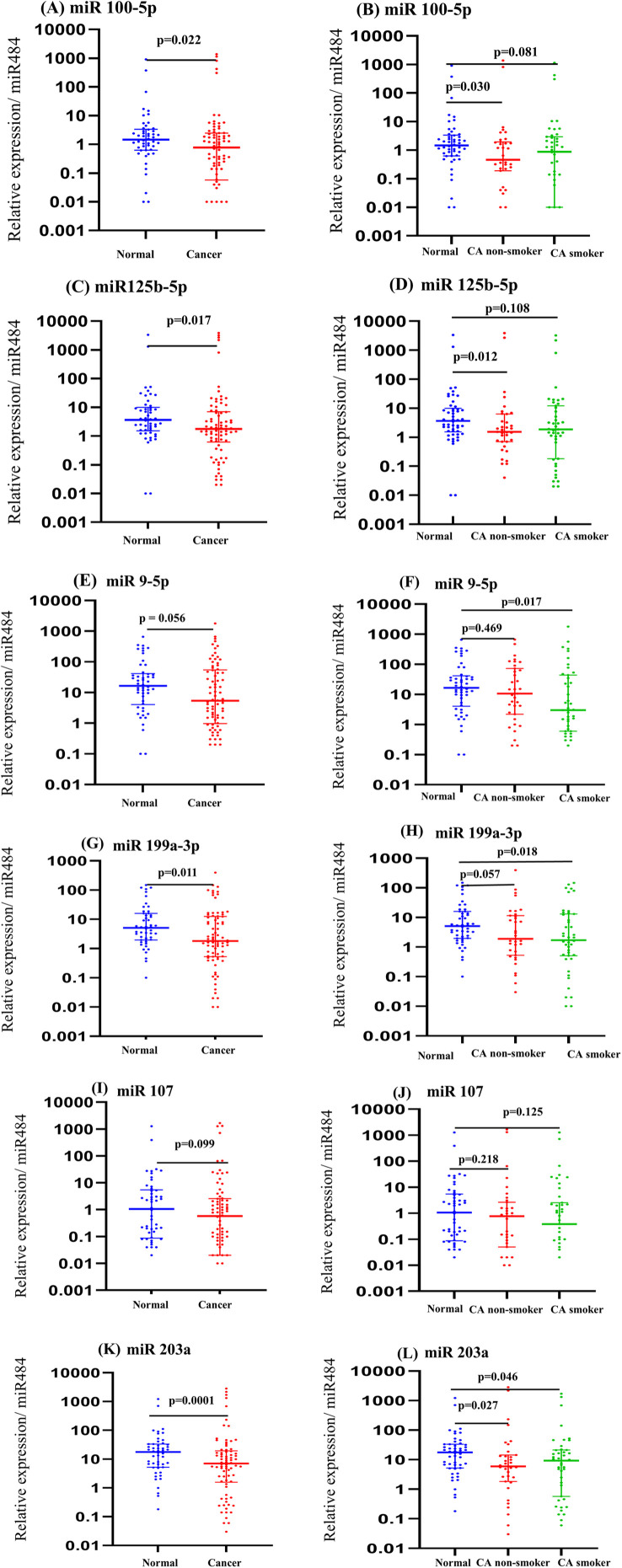
Scatter plots illustrating the serum expression levels of selected miRNAs validated by RT-qPCR in an expanded sample cohort. Figs 4a, 4c, 4e, 4g, 4i, and 4k compare NSCLC patients and non-cancer controls, while Figs 4b, 4d, 4f, 4h, 4j, and 4l further stratify NSCLC patients based on smoking history (CA non-smokers and CA smokers) in comparison to non-cancer controls. RT-qPCR validation was performed to confirm differential expression patterns identified from deep sequencing data. Data are presented as median with interquartile range (IQR), and statistical significance was assessed using the Mann-Whitney U test. The Y-axis is displayed on a log scale, and p-values indicate statistical significance for each comparison.

The results revealed a significant decrease in the expression levels of miR-100-5p and miR-125b-5p among overall NSCLC cancer patients (p = 0.022 and p = 0.017, respectively; Fig 4a, 4c). However, upon further classification based on smoking status, these two miRNAs were predominantly downregulated in NSCLC patients with no smoking history (p = 0.030 for miR-100-5p and p = 0.012 for miR-125b-5p; Fig 4b, 4d), while no significant difference was observed in NSCLC patients with smoking history (p = 0.081 and p = 0.108, respectively). For miR-9-5p, which was identified as significantly different in lung cancer smokers according to deep sequencing data, the RT-qPCR results confirmed a significant reduction in lung cancer smokers (p = 0.017; Fig 4f). However, although deep sequencing data suggested an increase in miR-9-5p expression, RT-qPCR results demonstrated a reduction, emphasizing the need for further validation. Regarding miR-203-3p, miR-107, and miR-199a-3p, which were selected due to their consistent downregulation in NSCLC patients, regardless of smoking status, RT-qPCR results confirmed a significant reduction in miR-203-3p and miR-199a-3p expression levels in both NSCLC patients with and without smoking history (p = 0.046 and p = 0.018, respectively; Figs 4h, 4l). However, miR-107 did not show a significant difference in either in entire NSCLC patients (p = 0.099, Fig 4i) or stratified according to history (p = 0.218 and p = 0.125, respectively; Figs 4j). These findings further support the potential role of miR-203-3p and miR-199a-3p as biomarkers for NSCLC independent of smoking history.

In addition, to further validate these associations and ensure they were independent of potential confounders, we performed multivariable binary logistic regression analyses including age, sex, and smoking status (Table 3). The results confirmed that miR-100-5p (OR = 0.66, 95% CI: 0.46–0.95, p = 0.026), miR-199a-3p (OR = 0.57, 95% CI: 0.35–0.92, p = 0.023), and miR-203a (OR = 0.62, 95% CI: 0.39–0.98, p = 0.042) were significantly associated with reduced odds of cancer after adjustment. miR-125b-5p showed a borderline association (OR = 0.68, 95% CI: 0.46–1.01, p = 0.060), while miR-9-5p and miR-107 did not demonstrate significant associations after adjustment. Smoking status remained the strongest independent predictor of cancer (OR ~8–9, p < 0.001), underscoring the importance of adjusting for this factor when evaluating circulating miRNAs as biomarkers.

**Table 3:** Multivariable binary logistic regression analysis of circulating miRNAs and cancer status, adjusted for age, sex, and smoking status.

miRNA	B (Coef.)	SE	Wald χ²	p-value	OR (Exp(B))	95% CI (approx.)
miR-125b-5p	−0.387	0.206	3.53	0.060	0.68	0.46–1.01
miR-100-5p	−0.414	0.186	4.94	0.026	0.66	0.46–0.95
miR-9-5p	−0.279	0.213	1.72	0.189	0.76	0.49–1.16
miR-199a-3p	−0.556	0.244	5.18	0.023	0.57	0.35–0.92
miR-107	−0.220	0.146	2.27	0.132	0.80	0.59–1.09
miR-203a	−0.478	0.235	4.14	0.042	0.62	0.39–0.98

Regression coefficient (B) represents the estimated change in the log odds of cancer per unit change in miRNA expression. Standard error (SE) indicates the variability of the coefficient estimate. Wald χ² is the Wald chi-square statistic used to test the significance of each predictor. p-value reflects the probability of observing the result under the null hypothesis. Odds ratio (OR, Exp(B)) represents the odds of cancer associated with one-unit change in miRNA expression. 95% confidence interval (CI) provides the range within which the true OR is expected to fall with 95% certainty. Negative B values and OR < 1 indicate lower expression in cancer patients relative to controls. Significant associations were observed for miR-100-5p, miR-199a-3p, and miR-203a, while miR-125b-5p showed a borderline association and miR-9-5p and miR-107 were not significant after adjustment.

To assess the potential impact of sex on miRNA expression, we conducted subgroup analyses within both control and lung cancer groups and the scatter plots are shown in the S5 Fig. Most miRNAs showed no statistically significant differences between male and female participants (all p-values > 0.05), except for miR-9-5p, which showed a significant decrease in male cancer patients (p = 0.035). Given the higher prevalence of smoking among male cancer patients in our cohort, this difference may reflect the influence of tobacco exposure rather than sex itself. These findings suggest that sex does not substantially influence the expression of the majority of miRNAs in this study.

Altogether, this validation confirms that miR-100-5p and miR-125b-5p are downregulated in non-smoker NSCLC patients, while miR-9-5p is specifically reduced in smokers, likely reflecting tobacco exposure. miR-203a-3p and miR-199a-3p were consistently downregulated regardless of smoking status, supporting their role as general NSCLC biomarkers. Overall, sex had minimal impact on miRNA expression

### 3.3. Chronic exposure to urban particulate matter (UPM) induced reduction of miR100-5p and miR125b-5p in primary bronchial/tracheal epithelial cells

To investigate the impact of particulate matter on the expression of miRNAs identified as significantly altered in lung cancer patients with no smoking history of smoking, primary bronchial/tracheal epithelial cells (HBECs) purchased from ATCC were treated with 50 and 100 ng/mL of urban PM for 1, 3, and 7 days, followed by quantification using RT-qPCR.

**Fig 5:**
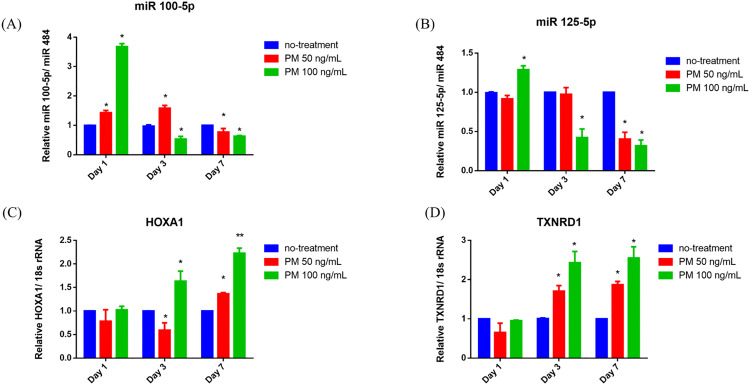
Expression levels of miR-100-5p (a) and miR-125b-5p (b), along with their respective target genes, *HOXA1* (c) and *TXNRD1* (d), in primary bronchial/tracheal epithelial cells (HBECs) exposed to urban particulate matter (UPM; SRM 1648a) for 1, 3, and 7 days. Expression levels are normalized to miR-484 for miRNA analysis and 18s rRNA for gene expression. The Y-axis represents Relative expression/ miR-484 for miRNAs and Relative expression/ 18s rRNA for target genes. Data represents the mean ± standard deviation (SD) from three independent experiments, each performed in triplicate (n = 9). Statistical significance was determined using the Mann-Whitney U test, with p < 0.05 indicated by (*).

Fig 5 illustrates that after 1-day exposure, miR-100-5p levels were notably increased (4.0-fold, p < 0.05 for 100 ng/mL PM), whereas miR-125b-5p showed no significant change. However, with prolonged exposure (3 and 7 days), both miRNAs exhibited a progressive and significant decrease in expression (miR-100-5p: 0.5-fold, p < 0.05 at day 7; miR-125b-5p: 0.2-fold, p < 0.05 at day 7). Analysis of culture supernatants collected after PM exposure also showed a transient rise in miR-100-5p and miR-125b-5p at 1 day, followed by a significant decline at 3 days and 7 days, mirroring the intracellular pattern (S8 Fig). This suggests that while acute exposure may transiently induce miR-100-5p expression, prolonged exposure to PM leads to sustained suppression of both miRNAs, potentially impairing their regulatory functions in lung epithelial cells.

Additionally, *TXNRD1* and *HOXA1* were selected as target genes of miR-125b-5p and miR-100-5p, respectively, based on previous studies demonstrating their regulation by these miRNAs [27,28] and their involvement in mTOR signaling [29,30], a key pathway enriched in our analysis (Fig 2b). Their expression patterns exhibited an inverse trend following PM exposure. After 1 day, *TXNRD1* and *HOXA1* expression levels slightly decreased, but significantly increased at 7 days (*HOXA1*: 2.2-fold, p < 0.05; *TXNRD1*: 2.7-fold, p < 0.05 at 100ng/mL urban PM). This suggests a time-dependent suppressive effect of PM exposure on miRNA expression, leading to de-repression of their target genes over time. These findings provide a mechanistic link between PM-induced miRNA dysregulation and activation of mTOR-related pathways, potentially contributing to lung cancer progression in non-smokers.

### 3.4. Effect of PM exposure on growth and metastatic potential of lung cancer cells

**Fig 6:**
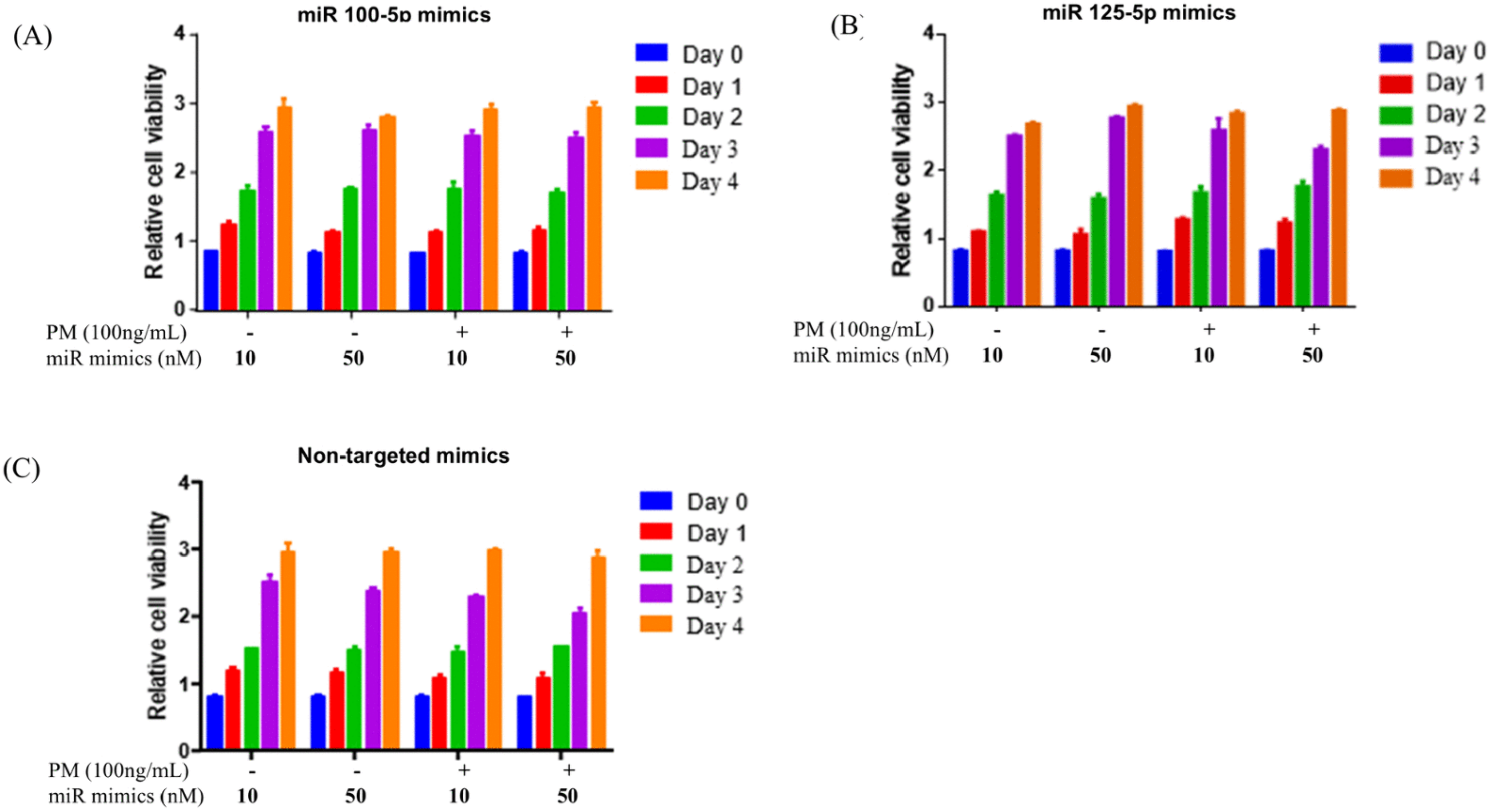
Relative cell viability of A549 cells treated with miR100-5p mimics (A), miR125b-5p mimics (B), or non-targeted mimics (C) at 10 nM and 50 nM, with or without 1.0 µg/mL PM exposure, over four days. No significant impact on proliferation was observed for miR100-5p or miR125b-5p mimics compared to controls. Data represents the mean ± standard deviation (SD) from three independent experiments, each performed in triplicate (n = 9).

This study investigated the effects of miR100-5p and miR125b-5p mimics on the growth of A549 lung cancer cells under both normal conditions and stimulation by urban particulate matter (PM), using non-targeted mimics as controls. Fig 6 illustrates the effects of miR100-5p and miR125b-5p mimics on A549 cell proliferation under particulate matter (PM) exposure over four days. As shown in Figs 6A and 6B, neither miR100-5p nor miR125b-5p mimics significantly influenced A549 cell proliferation, regardless of PM exposure, with proliferation patterns comparable to non-targeted controls (Fig 6C). Both concentrations (10 nM and 50 nM) of miRNA mimics failed to produce a noticeable difference, indicating that these miRNAs do not regulate proliferation under the tested conditions. It should be noted that the MTT assay used here primarily reflects cellular metabolic activity, which may not directly correspond to absolute changes in cell proliferation.

**Fig 7:**
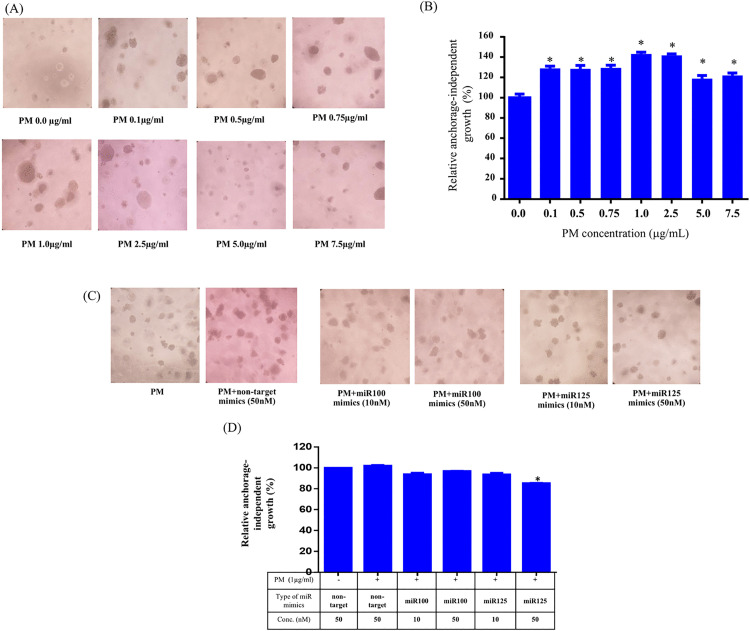
Effects of PM and miRNA mimics on the anchorage-independent growth of A549 cells. **(A)** Anchorage-independent growth of A549 cells treated with increasing concentrations of PM (0.1 to 7.5 µg/mL). Colony formation increased dose-dependently up to 2.5 µg/mL, indicating enhanced malignant behavior, but higher concentrations (5.0–7.5 µg/mL) were toxic and reduced colony growth. **(B)** Quantification of colony formation, showing a peak at 2.5 µg/mL, followed by a decrease at 5.0–7.5 µg/mL. **(C)** Representative images of colonies after treatment with PM (1.0 µg/mL) alone or with miRNA mimics (10 nM, 50 nM). **(D)** Quantification of **(C)**, showing a significant reduction (*p < 0.05) with 50 nM miR125b-5p mimics. Data represents the mean ± standard deviation (SD) from three independent experiments, each performed in triplicate (n = 9). Statistical significance was determined using the Mann-Whitney U test, with p < 0.05 indicated by (*).

The anchorage-independent growth assay demonstrated that PM significantly enhanced the colony-forming ability of A549 cells, supporting its role in promoting malignant behavior. Fig 7A shows representative images of soft agar colonies formed at different PM concentrations, indicating a dose-dependent increase in colony formation from 0.1 to 2.5 µg/mL. At higher PM concentrations (5.0–7.5 µg/mL), the colony formation slightly decreased compared to peak levels but remained above the non-treated control, suggesting a reduced stimulatory effect. Fig 7B quantifies these findings, illustrating the trend of increased anchorage-independent growth at lower PM concentrations. Based on these observations, a PM concentration of 1.0 µg/mL was selected for subsequent experiments involving miRNA mimic treatments.

Fig 7C demonstrates that miR125b-5p mimics suppressed PM-induced colony formation in a dose-dependent manner, with the 50 nM concentration showing the most pronounced effect. Quantitative analysis in Fig 7D confirms this suppression, with miR125b-5p mimics (50 nM) achieving a significant reduction in colony formation (fold change = 0.85, p < 0.05) compared to PM-only and non-targeted controls.

**Fig 8:**
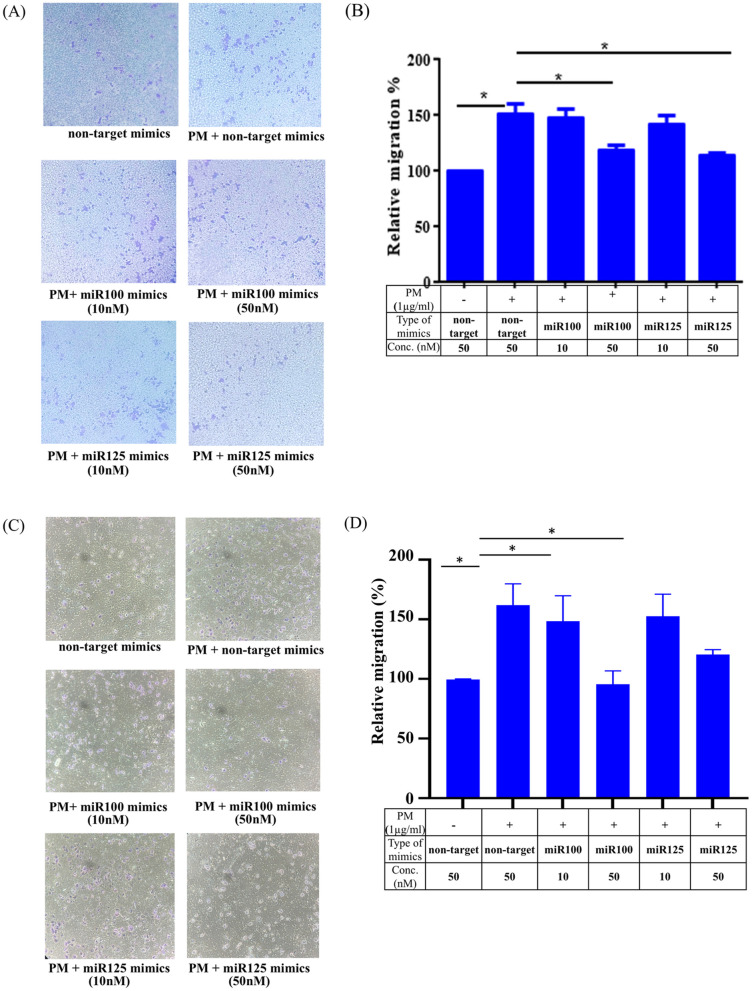
Effects of PM and miRNA mimics on migration of A549 cells. (A) and **(C)** Representative images of migrated A549 and H1299 cells, respectively, under various conditions: non-target mimics, PM + non-target mimics, PM + miR-100 mimics (10 nM and 50 nM), and PM + miR-125 mimics (10 nM and 50 nM). miRNA mimics were transfected first, followed by PM (1.0 µg/mL) treatment immediately after transfection. Cells were incubated for 20 hours before migration analysis. (B) and **(D)** Quantitative analysis of relative migration (fold change) of migrated A549 and H1299 cells, respectively. PM exposure significantly increased cell migration, and miR-100/miR-125 mimics exhibited dose-dependent effects. Data represent the mean ± standard deviation (SD) from three independent experiments, each performed in triplicate (n = 9). Statistical significance was determined using the Mann-Whitney U test, with p < 0.05 indicated by (*).

To investigate the effect of miRNA mimics on PM-induced cell migration, a transwell migration assay was performed. As shown in Fig 8A, PM treatment significantly enhanced cell migration compared to the non-targeted mimics. However, co-treatment with miR100-5p or miR125b-5p mimics resulted in a noticeable reduction in migrated cells, with the inhibitory effect being more pronounced at the 50 nM concentration. Quantitative analysis further confirmed these observations (Fig 8B). PM treatment increased cell migration by approximately 1.5-fold compared to the non-targeted mimics (p < 0.05). In contrast, cells treated with miR100-5p mimics at 10 nM and 50 nM reduced migration to approximately 1.3-fold and 1.2-fold, respectively, relative to the PM-treated control (p < 0.05). Similarly, miR125b-5p mimics at 10 nM and 50 nM decreased migration to approximately 1.3-fold and 1.1-fold, respectively, compared to the PM-treated control (p < 0.05). The strongest inhibitory effect was observed with the 50 nM miR125b-5p mimic. These findings suggest that miR100-5p and miR125b-5p play a critical role in counteracting the pro-migratory effects of PM exposure in A549 cells. Given the central role of cell motility in cancer progression, we additionally examined this assay in H1299 cells to confirm the generalizability of the findings. Consistent with the results in A549, PM significantly increased cell migration in H1299 cells, while both miR-100-5p and miR-125b-5p mimics dose-dependently reduced PM-induced migration (Figs 8C–D), with the 50 nM miR-125b-5p mimic again demonstrating the strongest inhibitory effect.

## Discussion

Despite the decline in lung cancer mortality attributed to tobacco control measures, a significant proportion of cases still occur in non-smokers. Understanding non-smoking-related lung cancer is crucial for developing new interventions and reducing the global lung cancer burden. Approximately 25% of lung cancer cases worldwide occur in individuals who have never smoked, with regional variation noted, such as less than 20% in the U.S [31] but can exceed 40% in regions like Asia and Africa [32]. The shift has intensified the focus on non-tobacco-related causes, particularly environmental factors such as radon gas and air pollution, as potential contributors to lung cancer in non-smokers. miRNAs serve as molecular fingerprints of environmental impact on cells due to their role in regulating gene expression. Air pollutants can alter miRNA expression patterns within cells, reflecting the cell’s response to its environment and serving as biomarkers for environmental exposures. To ensure accurate quantification of circulating miRNAs, careful selection of an appropriate endogenous control is essential. In this study, we evaluated several reference candidates, including miR-361-5p [33], U6 snRNA [34], and miR-484 [35,36], and selected miR-484 based on its stable expression across cancer and control serum samples in our preliminary analysis.

Our findings provide evidence of the association between PM exposure and non-smoking-related lung cancer in Northern Thailand. Through RNA sequencing of circulating miRNAs, we compared lung cancer patients with and without a smoking history to non-cancer controls, revealing a distinct set of miRNAs, including miR125b-5p and miR100-5p, with decreased expression level specifically in non-smoker lung cancer cases. In our study design, the non-cancer control group included both smokers and non-smokers, ensuring that any smoking-related alterations in miRNA expression were inherently represented within the baseline reference. This approach allowed us to isolate lung cancer-specific miRNA changes while accounting for potential confounding effects of smoking. The consistent downregulation of miR-100-5p and miR-125b-5p in non-smoking lung cancer cases, along with their milder reduction in some smokers, suggests that these miRNAs may primarily reflect PM-associated lung cancer risk, with smokers potentially experiencing a combined impact of both cigarette smoke and PM exposure. Further investigation showed that the expression of miR-125b-5p and miR-100-5p was initially elevated in response to urban particulate matter, indicating a stress response. However, chronic exposure for 3–7 days resulted in the suppression of these miRNAs. This was in contrast to their target mRNA expressions (*TXNRD1* and *HOXA1*), thus providing a molecular link between air pollution and non-smoker lung cancer. Consistent with these observations, analysis of publicly available TCGA miRNA profiles confirmed lower tumor expression of miR-100-5p and miR-125b-5p, providing external support for our serum findings and their potential use as circulating markers of PM-associated lung cancer risk (S7 Fig)

Both miR-100-5p and miR-125b-5p have well-documented anti-tumor properties, functioning as tumor suppressors in various cancers [37–40]. miR-100-5p inhibits cell proliferation, migration, and invasion, as well as signaling pathways such as Wnt/β-catenin, particularly in non-small cell lung cancer (NSCLC), where it targets *HOXA1* to suppress tumor progression and epithelial-mesenchymal transition (EMT) [23,37,41–47]. Similarly, miR-125b-5p demonstrates tumor-suppressive roles by targeting TNFR2, reducing regulatory T-cell immunosuppression, and enhancing immune responses. Reduced expression of miR-125b-5p in lung adenocarcinoma correlates with poor prognosis, while its overexpression suppresses cell proliferation, migration, and invasion and induces apoptosis [22,48,49]. Notably, miR-125b-5p exhibits context-dependent functions, including oncogenic roles in specific cancers [50].

The biphasic expression pattern of miR-100-5p and miR-125b-5p following PM exposure likely reflects distinct phases of cellular stress response. After acute exposure (Day 1), both miRNAs are upregulated, suggesting an early adaptive mechanism. miR-100-5p, known for its role in stress adaptation via mTOR suppression, promotes autophagy to mitigate oxidative and endoplasmic reticulum (ER) stress, as observed in cardiac hypertrophy [51]. Similarly, miR-125b-5p, a key regulator of inflammatory responses may initially increase to activate immune response, aligning with its role in acute inflammation in pancreatitis [52]. However, after prolonged exposure (Day 3 and 7), their expression declines, likely due to stress exhaustion and cellular dysfunction. Chronic PM exposure may lead to excessive ER stress and mTOR inhibition, impairing autophagy and cellular recovery, which could explain the reduction in miR-100-5p. Meanwhile, the decline in miR-125b-5p may reflect a transition from acute inflammation to immune suppression or chronic low-grade inflammation.

To further explore the implications of our findings, we conducted functional studies using transfection experiments with miR-125b-5p and miR-100-5p mimics in lung cancer cell lines. The results demonstrated that restoring the expression of these miRNAs effectively suppressed PM-induced cancer cell mobility and colony formation, two critical hallmarks of cancer progression. Specifically, transfection with miR-125b-5p mimics significantly reduced both PM-induced cancer cell mobility and colony formation, whereas miR-100-5p mimics primarily inhibited cancer cell mobility. These findings have significant implications for cancer prevention strategies, particularly in high-risk populations exposed to elevated levels of air pollution. miR-125b-5p and miR-100-5p could serve as potential biomarkers for early detection of environmental exposure-related lung cancer risk.

Furthermore, therapeutic interventions aimed at restoring or mimicking the function of these miRNAs might provide a novel approach to mitigate the adverse effects of air pollution on lung health. This mechanistic validation revealed consistent dysregulation of miR-125b-5p and miR-100-5p within the cellular environment, reinforcing that the changes observed in serum reflect tumor-intrinsic processes rather than solely systemic influences. our Previous studies have shown that a subset of miRNAs consistently exhibits concordant dysregulation patterns in both tumor tissues and circulating serum, highlighting their potential as non-invasive biomarkers with mechanistic relevance [53,54]. The key lies in identifying the right candidates with stable and disease-representative expression [55]. Nevertheless, our intracellular validation strengthens the hypothesis that miR-125b-5p and miR-100-5p are functionally involved in lung cancer pathogenesis and may serve as relevant biomarkers for pollution-associated lung cancer.Our study has also highlighted miR203a and miR199a-3p as specific biomarkers for lung cancer irrespective of smoking status. MiR-203a plays a pivotal role in lung cancer, being significantly downregulated in non-small cell lung cancer (NSCLC). Interestingly, its expression can be augmented by low-temperature plasma treatment, leading to the inhibition of cell growth and induction of apoptosis in lung cancer cells [56]. Moreover, miR-203a targets genes like AVL9 and BIRC5, influencing cell proliferation, migration, and invasion in NSCLC [57]. Its presence in circulating biofluids like serum underscores its potential as a non-invasive biomarker for lung cancer detection and monitoring [58]. Additionally, extracellular vesicle-derived miR-203a from human umbilical vein endothelial cells has been found to suppress malignant behaviours of NSCLC cells by targeting DTL and promoting p21 protein stability [59]. These findings underscore miR-203a’s potential as a diagnostic, prognostic, and therapeutic target in lung cancer, providing valuable insights into its role in disease progression and treatment.

Similarly, in lung adenocarcinoma (LUAD), miR-199a-3p targets anterior gradient 2 (AGR2), suppressing tumorigenesis and promoting apoptosis [60]. Additionally, it enhances the sensitivity of NSCLC to gefitinib, an EGFR-T790M inhibitor, suggesting its potential as a therapeutic target and diagnostic marker for NSCLC [61]. These miRNAs represent promising biomarkers for identifying lung cancer, awaiting further validation.

In summary, despite the decline in lung cancer mortality due to tobacco control measures, a significant number of cases still occur in non-smokers, necessitating the exploration of non-tobacco-related causes such as environmental factors. Our study provides evidence linking PM exposure to non-smoking-related lung cancer in Northern Thailand, identifying distinct miRNA expression patterns, including miR-125b-5p and miR-100-5p, associated with air pollution. These miRNAs, known for their tumor-suppressive functions, exhibited decreased expression in non-smoker lung cancer cases. Further research revealed that air pollutants could alter miRNA expression, serving as biomarkers for environmental exposures. Additionally, miR-203a and miR-199a-3p emerged as potential biomarkers for lung cancer, regardless of smoking status, highlighting their roles in disease progression and treatment. These findings underscore the complex interplay between miRNAs, environmental factors, and lung cancer, offering new insights for future diagnostic, prognostic, and therapeutic strategies.

## Supporting information

S1 Data Raw data for Fig 5. This file contains the complete dataset used to generate Fig 5, including relative expression levels and all statistical analyses. (XLSX)

S2 Data Raw data for Fig 6. This file includes all values used in Fig 6, covering PM-dose and time-course responses. (XLSX)

S3 Data Raw data for Fig 7. This dataset contains all measurements used in Fig 7, including experimental replicates and normalized miRNA expression values. (XLSX)

S4 Data Raw data for Fig 8. This file contains the full dataset for Fig 8, including RT-qPCR values from culture supernatants at days 1, 3, and 7 after PM exposure. (XLSX)

S5 Fig Distribution of investigated miRNAs between genders. This figure presents circulating miRNA levels in male and female healthy controls and lung cancer patients.
(PDF)

S6 Data Basal expression levels of miR-100-5p and miR-125b-5p. This spreadsheet contains baseline expression values and associated clinical metadata. (XLSX)

S7 Fig Expression of miR-100-5p and miR-125b-5p in TCGA LUAD and LUSC datasets stratified by smoking history. This figure shows expression patterns of miR-100-5p and miR-125b-5p across smoking-status groups in LUAD and LUSC TCGA cohorts.
(PDF)

S8 Fig Secreted miRNAs after PM exposure. RT-qPCR measurements of miR-100-5p and miR-125b-5p in culture supernatants at 1, 3, and 7 days following PM exposure.
(PDF)
