# Supplementary material for: MiR-125b-5p and miR-100-5p as Biomarkers and therapeutic targets for the prevention of particulate matter-induced non-smoker lung cancer
Source: PLoS One. 2025 Dec 2;20(12):e0337805. doi: 10.1371/journal.pone.0337805 (PMC12671819; doi:10.1371/journal.pone.0337805)
Supplement: S5 Fig — This figure presents circulating miRNA levels in male and female healthy controls and lung cancer patients. (PDF) [file pone.0337805.s005.pdf]

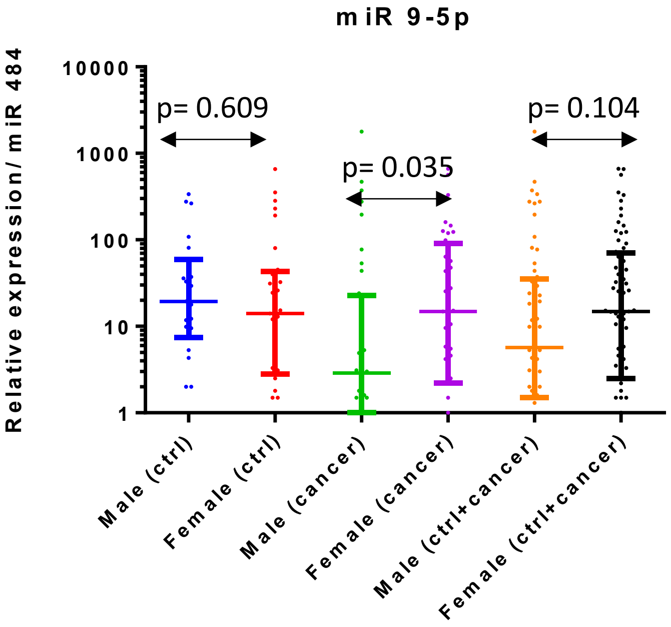


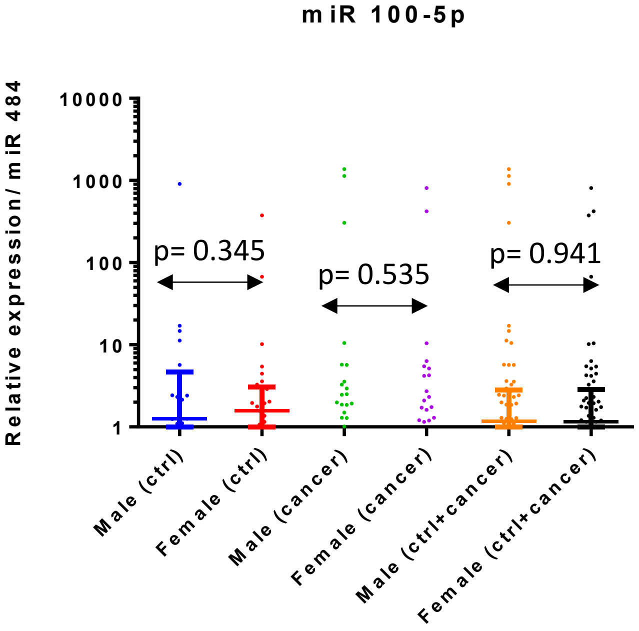


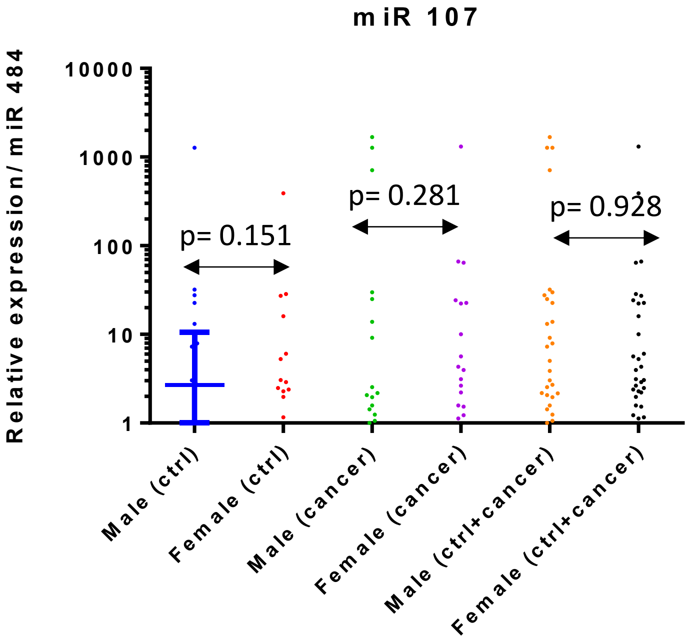


Male (ctrl)= 21

Female (ctrl)= 29

Male (cancer)= 39

Female (cancer)= 39

Male (ctrl+cancer)= 60

Female (ctrl+cancer)= 68

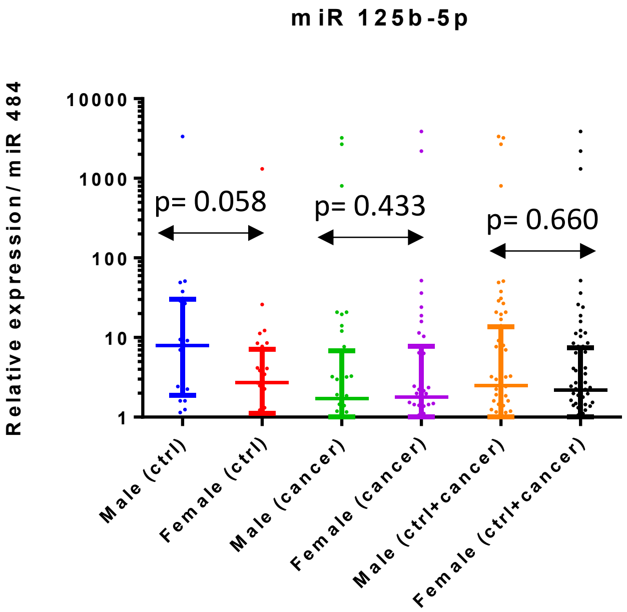

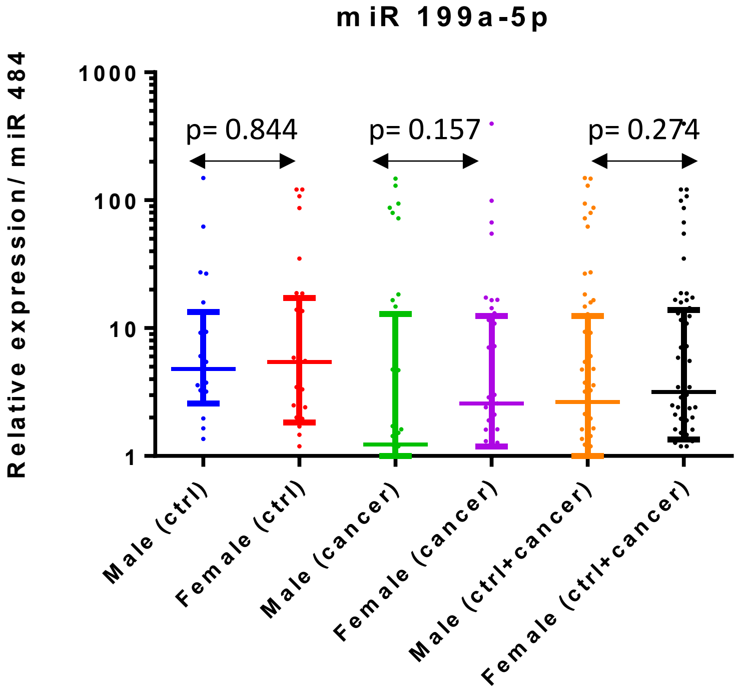


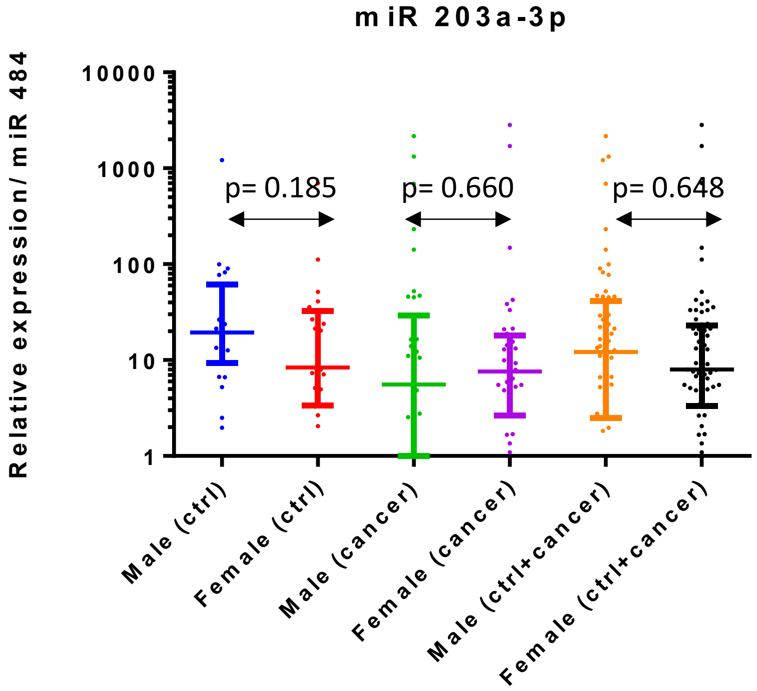


Male (ctrl)= 21

Female (ctrl)= 29

Male (cancer)= 39

Female (cancer)= 39

Male (ctrl+cancer)= 60

Female (ctrl+cancer)= 68
