# Supplementary material for: MiR-125b-5p and miR-100-5p as Biomarkers and therapeutic targets for the prevention of particulate matter-induced non-smoker lung cancer
Source: PLoS One. 2025 Dec 2;20(12):e0337805. doi: 10.1371/journal.pone.0337805 (PMC12671819; doi:10.1371/journal.pone.0337805)

Supplementary Figure S7.

Expression of miR-100-5p and miR-125b-5p in TCGA LUAD and LUSC datasets stratified by smoking history.

Level-3 miRNA-sequencing data and corresponding clinical information for lung adenocarcinoma (LUAD) and lung squamous cell carcinoma (LUSC) were obtained from The Cancer Genome Atlas (TCGA) (https://portal.gdc.cancer.gov. Statistical analyses were performed using R software (version 4.0.3). Box-and-dot plots display the expression distribution of miR-100-5p and miR-125b-5p across tumor samples from patients with or without a smoking history and in normal lung tissues. The x-axis represents sample groups, and the y-axis represents normalized miRNA expression levels. Statistical significance was determined using the Kruskal–Wallis test. Asterisks denote *p*-value thresholds: *p* < 0.05 (*), *p < 0.01 (**), p < 0.001 (***)*, and *p* < 0.0001 (****).

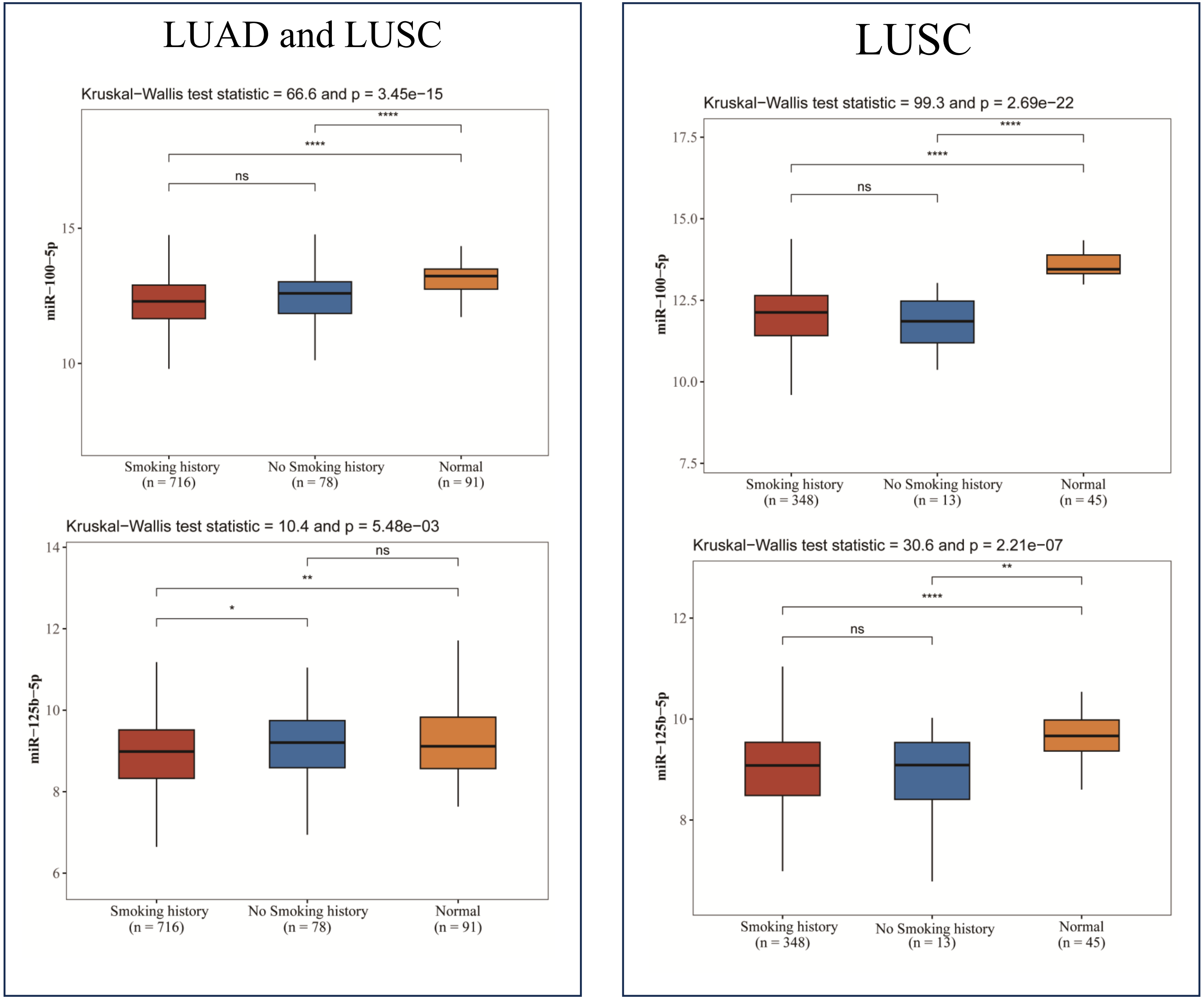

Supplement: S7 Fig — This figure shows expression patterns of miR-100-5p and miR-125b-5p across smoking-status groups in LUAD and LUSC TCGA cohorts. (PDF) [file pone.0337805.s007.pdf]
