# Supplementary material for: MiR-125b-5p and miR-100-5p as Biomarkers and therapeutic targets for the prevention of particulate matter-induced non-smoker lung cancer
Source: PLoS One. 2025 Dec 2;20(12):e0337805. doi: 10.1371/journal.pone.0337805 (PMC12671819; doi:10.1371/journal.pone.0337805)
Supplement: S8 Fig — RT-qPCR measurements of miR-100-5p and miR-125b-5p in culture supernatants at 1, 3, and 7 days following PM exposure. (PDF) [file pone.0337805.s008.pdf]

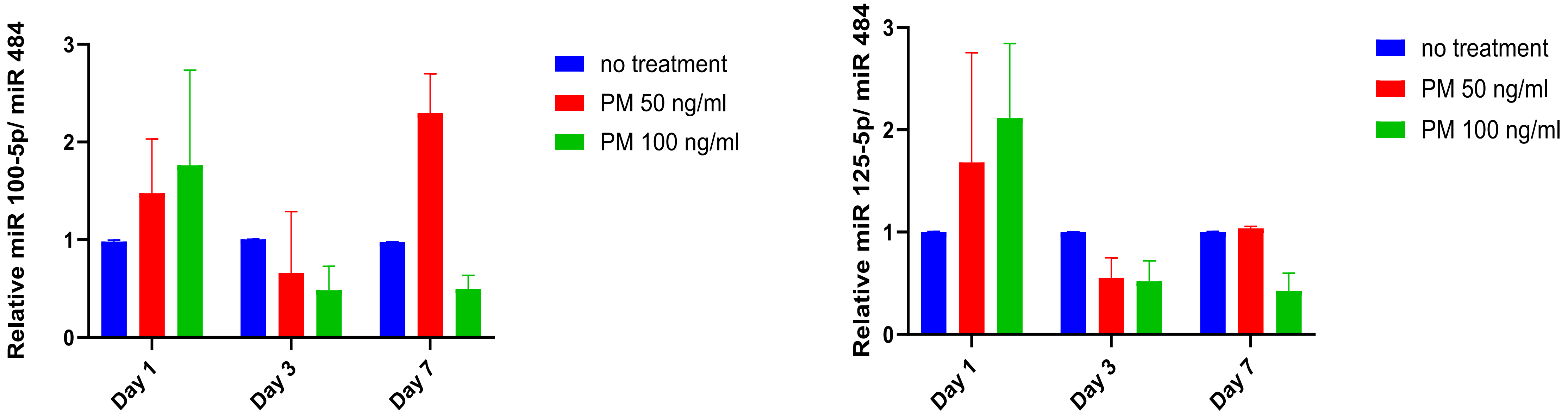

Supplementary data Figure S8 Secreted miRNAs after PM exposure. RT-qPCR of miR-100-5p and miR-125b-5p in culture supernatants at 1, 3, and 7 days following 50 or 100 ng/mL PM. Bars show mean ± SD; normalization to miR-484. Both miRNAs increase at 24 h and decline with prolonged exposure, consistent with intracellular measurements
